# Nanoparticulate Poly(glucaramide)-Based Hydrogels for Controlled Release Applications

**DOI:** 10.3390/gels3020017

**Published:** 2017-05-06

**Authors:** Erik R. Johnston, Tyler N. Smith, Monica A. Serban

**Affiliations:** 1 Materials Science Program, University of Montana, 32 Campus Dr., Missoula, MT 59812, USA; erik.johnston@umconnect.umt.edu (E.R.J.); tyler@rivertop.com (T.N.S.); 2 Rivertop Renewables, 1121 E Broadway St # 135, Missoula, MT 59802, USA; 3 Department of Biomedical and Pharmaceutical Sciences, University of Montana, 32 Campus Dr., Missoula, MT 59812, USA

**Keywords:** d-Glucaric acid, polymers, nanoparticles, hydrogel, controlled release

## Abstract

In 2004, d-Glucaric acid (GA) was identified as one of the top value-added chemicals from renewable feedstocks. For this study, a patented synthetic method was used to obtain gel forming polymers through the polycondensation of GA and several aliphatic diamines. The first time characterization and a potential practical application of such hydrogels is reported herein. Our findings indicate that the physical properties and gelling abilities of these materials correlate with the chemical structure of the precursor diamines used for polymerization. The hydrogels appear to have nanoparticulate nature, form via aggregation, are thermoresponsive, and appear suitable as controlled release systems for small molecules. Overall, this study further highlights the versatility of GA as a building block for the synthesis of sustainable materials, with potential for a wide array of applications.

## 1. Introduction

Hydrogels obtained via various methods have been extensively characterized and used in applications ranging from tissue engineering, to controlled release systems, and super-absorbent materials [1,2,3,4,5]. Their vast array of usability results from their ability to absorb and retain proportionally large quantities of water, versatile mechanical and physico-chemical properties, and, when used in biomedical applications, their excellent biocompatibility [1,6,7,8]. In addition, many of the viscoelastic properties of hydrogels can be tuned to respond to environmental stimuli, such as temperature, pH, ionic strength, etc. [7,9,10]. Mechanistically for a hydrogel to form, the gel or network forming substrate must assemble via a crosslinking mechanism. Crosslinking can occur through chemical, light-induced or physical assemblies, all of which have been widely explored [1]. Each approach has its advantages and disadvantages. Chemical and photo-crosslinking are typically rapid, result in the formation of covalent bonds between the gel forming constituents and allow for the tailoring of mechanical or controlled release properties of the hydrogel [11,12]. In contrast, physical crosslinking relies on hydrogen bonding, electrostatic interactions or other non-covalent phenomena to assemble the hydrogel network. Such hydrogels typically require a longer gelation period, and their mechanical and controlled release properties are less controllable [1,13].

In the context of glucaric acid-based materials, the hydroxyl and amide functional groups of the polymers provide ample opportunities for physical crosslinking interactions. The principal precursor, GA is commonly synthesized through the chemical or biological oxidation of d-glucose, a widely available monosaccharide produced from the hydrolysis of corn, potato, rice and other grain derived starches [14,15,16,17]. In a 2004 report, the US Department of Energy listed GA as one of the “Top Twelve Value Added Chemicals from Renewable Feedstocks”, due to its potential as a chelant and corrosion inhibitor, and as a precursor for the synthesis of sustainable materials [18]. An example of the latter are poly(glucaramide)s obtained via the condensation reaction between GA and virtually any primary diamine [19]. Certain GA/diamine combinations have been shown to form physically crosslinked hydrogels under select concentration, solvent and temperature conditions [20]. However, the physical and mechanical properties of these hydrogels as well as their gelling mechanism have not been explored. As a potential application area, the GA hydrogel materials were investigated as agents for the controlled release of small molecules, exemplified by sodium tripolyphopsphate (STPP) [21,22,23]. STPP is a commodity chemical widely employed in agricultural, industrial, and consumer products [21,24,25]. However, its agricultural use as nutrient in its current form has been associated with eutrophication of surface water—a detrimental environmental impact resulting from increased nutrient levels in aquatic ecosystems [26]. Our research shows that biodegradable poly(glucaramide)s have the ability to gel in the presence of high concentrations of STPP and then modulate its controlled release into the surrounding environment. The slow-release of STPP could allow for a localized and targeted utilization of the molecule and could potentially reduce the use of phosphorus and the inherent risks of eutrophication. Importantly, as indicated by previously published data, by-products from the degradation of poly(glucaramide)s constitute a source of nitrogen that can be utilized by crops [27]. In this specific application’s context, the dual benefit and synergistic nutritive potential of poly(glucaramide) hydrogels makes them attractive candidates as next generation sustainable nutrient delivery systems.

## 2. Results and Discussion

For this study, we focused on gel forming poly(glucaramide)s derived from 1,4-butanediamine and 1,6-hexanediamine. Both these diamines are widely available and are cost-effective relative to other counterparts.

### 2.1. Synthesis of Poly(glucaramide)s

**Figure 1:**
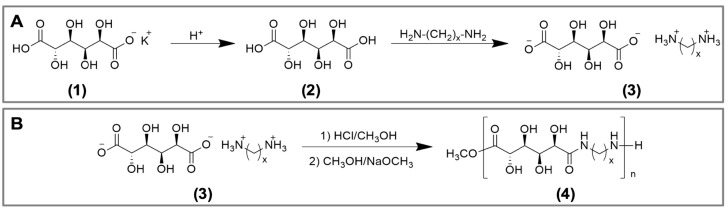
Syntheses of poly(glucaramide)s. (**A**) reaction scheme for the formation of polymerization precursors for the synthesis of poly(glucaramide)s. Monopotassium glucarate (1) is acidified to glucaric acid (2), which is then reacted with a diamine of choice to produce a diammonium d-glucarate salt (3) (**B**) reaction scheme for the synthesis of poly(glucaramide)s. Diammonium d-glucarate salts (3) are esterified in methanolic HCl then neutralized with sodium methoxide to form diamine poly(glucaramide)s (4) via polycondensation.

The poly(glucaramide)s were obtained as previously reported [20]. As a first step in polymers synthesis, monopotassium glucarate (1) was acidified to glucaric acid (2) then allowed to react with the desired diamine to produce a diammonium d-glucarate salt (3) (Figure 1A). This step ensures stoichiometric balance of the co-monomers. Next, the diammonium d-glucarate salt (3) was esterified in methanolic HCl and then neutralized with sodium methoxide to promote polycondensation to form single diamine poly(glucaramide)s (4) (Figure 1B). Alternatively, if two different diammonium d-glucarate salts are reacted, a mixed diamine poly(glucaramide) can be obtained. For this study, three different poly(glucaramide)s were employed. The first polymer, C4 was obtained from glucaric acid and tetramethylene diamine. The second polymer, C4C6—a mixed poly(glucaramide), was obtained from glucaric acid with tetramethylene diamine and glucaric acid with hexamethylene diamine. The third poly(glucaramide), C6 was obtained from glucaric acid and hexamethylene diamine. The structures and degrees of polymerization for the synthesized poly(glucaramide)s were confirmed by ^1^H-NMR (data not shown) as previously described [19].

### 2.2. Preparation of Hydrogels

**Figure 2:**
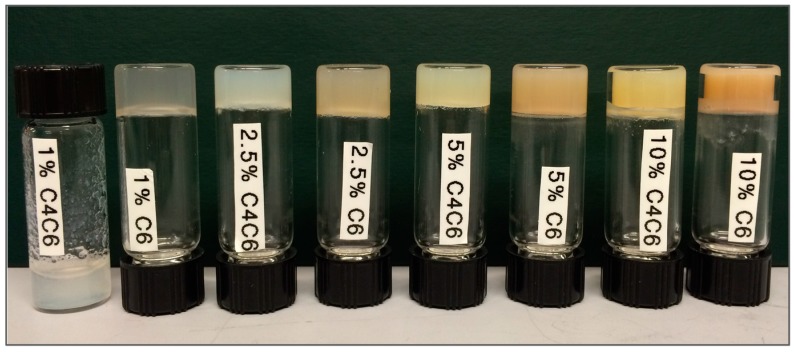
Physical appearance of poly(glucaramide) gels obtained from C4C6 and C6 polymer solutions, respectively, ranging from 1% *w/w* (**left**) to 10% *w/w* (**right**). C4C6 mixed poly(glucaramide)s formed gels at concentrations ≥2.5% *w/w*, while C6 polymers formed gels at concentrations ≥1% *w/w*. C4 polymer solutions did not form gels in the 1–10% *w/w* concentration range tested.

The three poly(glucaramide)s—C4, C4C6 and C6—were assessed for their hydrogel forming abilities. C4 polymer solutions did not form gels, regardless of the polymer concentration in solution (tested up to 10% *w/w*, data not shown). The C4C6 and C6 polymers yielded semi-transparent to opaque, white to yellow, softer to stiffer hydrogels (as visually assessed) in a concentration dependent manner (Figure 2). As with the gel forming ability, the minimum gelling concentration (MCG) was dependent on the diamine portion of the polymer, and was different for C4C6 and C6 polymer solutions. Specifically, the C4C6 polymer required concentrations greater than 1% solution for gel formation (Figure 2). The C6 polymer was capable of forming gels at 1% concentration; however, the polymer required a co-solvent, in this case acetic acid, to promote initial solubilization. A summary of the selected polymers water solubility and gel forming parameters is presented in Table 1.

### 2.3. Characterization of Hydrogels

#### 2.3.1. SEM Characterization

**Figure 3:**
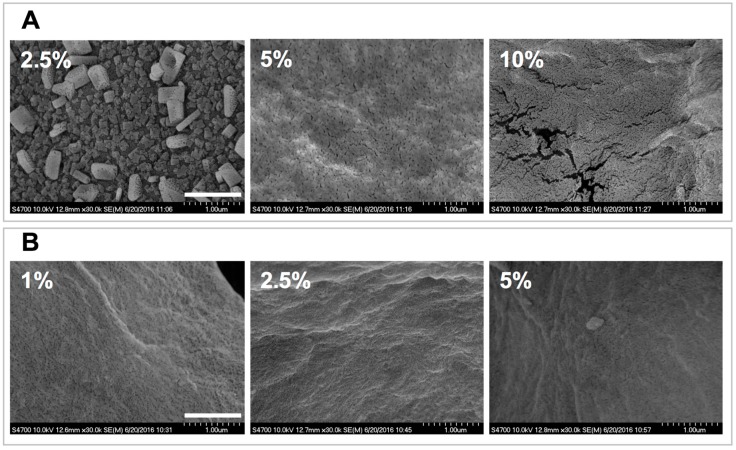
SEM analyses of poly(glucaramide) hydrogels. (**A**) micrographs of lyophilized 2.5%, 5% and 10% *w/w* C4C6 gels. For the 2.5% *w/w* C4C6 samples the structural hierarchy appears to indicate the formation of nanoparticle blocks (**top right**) while at higher concentrations the nanoparticulate nature of the samples appears more homogeneous; (**B**) micrographs of lyophilized 1%, 2.5% and 5% *w/w* C6 gels indicative of the nanoparticulate nature of the samples. The scale bar is 1 μm.

The structures of the polymeric networks were initially analyzed via SEM (Figure 3). The obtained micrographs indicate that the dehydrated gels have a particulate nature and that they appear to be formed via aggregation of nanoparticles. For the 2.5% C4C6 samples the structural hierarchy appears to indicate the formation of nanoparticle blocks (Figure 3A). In contrast, C6 materials do not display a similar aggregation pattern and the nanoparticle distribution appears more homogeneous (Figure 3B). The 10% C6 formulation was not investigated via SEM, even though a hydrogel was formed, as the starting solution appeared to contain undissolved polymer. 

#### 2.3.2. DLS Analyses

**Figure 4:**
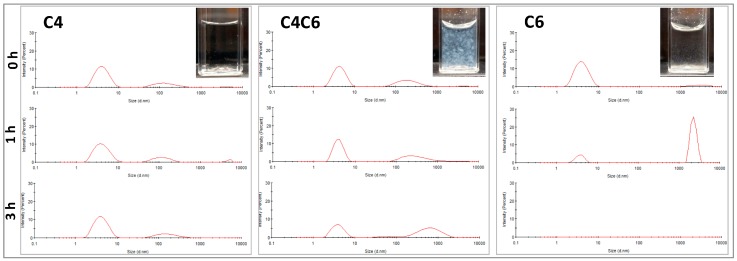
Particle analyses of the poly(glucaramide) solutions. Particle size distribution in water at 0, 1 and 3 h for the C4 (**left**), C4C6 (**middle**) and C6 (**right**) polymer solutions as observed by dynamic light scattering (DLS). All solutions were prepared at non-gelling concentrations (0.5% *w/w*) to allow the observation of the nanoparticles and their behavior in solution. Axes information: *x*—Intensity (%); scale: 0–30%; *y*—Size (d. nm); scale: 0–10,000 nm. Insets—Visual appearance of poly(glucaramide) solutions after 24 h illustrating the absence of aggegation or precipitation in C4 solutions (**top left**), aggregation and pre-gellation in C4C6 solutions (**top middle**) and precipitation in C6 solutions (**top right**).

Based on the SEM observations, the particulate appearance of the materials was further investigated to eliminate any potential SEM artifacts that could have occurred during sample preparation/hydrogel dehydration. In this context, dilute poly(glucaramide) solutions were analyzed by DLS at three different time points (Figure 4).

For all polymeric solutions, the concentrations investigated (≤0.5% *w/w*) were chosen to be below the minimum gelling concentration (MGC). The dynamic light scattering (DLS) data indicate that the C4 poly(glucaramide)s form stable nanoparticle suspensions with one predominant population in the ~4 nm diameter range and a second one in the ~150 nm diameter range (Figure 4 and Table 2). This distribution was maintained during the 3 h experimental time frame (Table 2). The C4C6 polymers elicited an initial nanoparticle distribution similar to their C4 counterparts, with a predominant (~66%) diameter size in the ~4 nm range (Figure 4 and Table 2). However, at 3 h the population percentage shifted predominantly to larger particle sizes in the range of ~700 nm (~54%), in parallel with a decrease of the ~4 nm diameter population to ~41%, indicative of aggregation (Table 2). Similarly, the C6 poly(glucaramide) solutions had an initial predominant population distribution in the ~4 nm range (~95%), that rapidly (within 1 h) shifted to larger diameters in the ~2000 nm range, indicative of an aggregation driven gelation mechanism. At 3 h, no nanoparticles in the window measureable by DLS were observed indicating another shift to larger particles (Table 2). It is important to note that, inherent to the technique, larger particles scatter more light than smaller particles. Therefore, the percentage intensities noted should not be correlated with particle numbers, but rather with overall particle populations. The visual assessment of the analyzed solutions at 24 h was consistent with the DLS data; the C4 solution remained clear, the C4C6 solution showed aggregation, while the C6 polymer solution formed particles that sedimented to the bottom of the cuvette (Figure 4). 

Taken together, the SEM and DLS data appear to suggest that the presence of the C6 diamine (which differs from C4 in its degree of hydrophobicity) is a driving factor for gel formation via a nanoparticle aggregation mechanism. In a broader sense, our data indicates that the proportion of hydrophobic to hydrophilic moieties in these poly(glucaramide)s are dictating their aqueous solubility and tendency to aggregate in larger nanoparticle clusters which drives hydrogel formation.

#### 2.3.3. Rheological Characterization

With an understanding of the hydrogel structures, the macroscopic properties of these materials were investigated rheologically. First, the viscoelastic region of these materials was examined to determine the appropriate oscillation stress for further testing (data not shown). The readings for both hydrogels (C4C6 and C6), and all tested concentrations (2.5–10% for C4C6 and 1–10% for C6), were linear in the 1–200 Pa range. Therefore, all further testing was conducted within these stress values.

**Figure 5:**
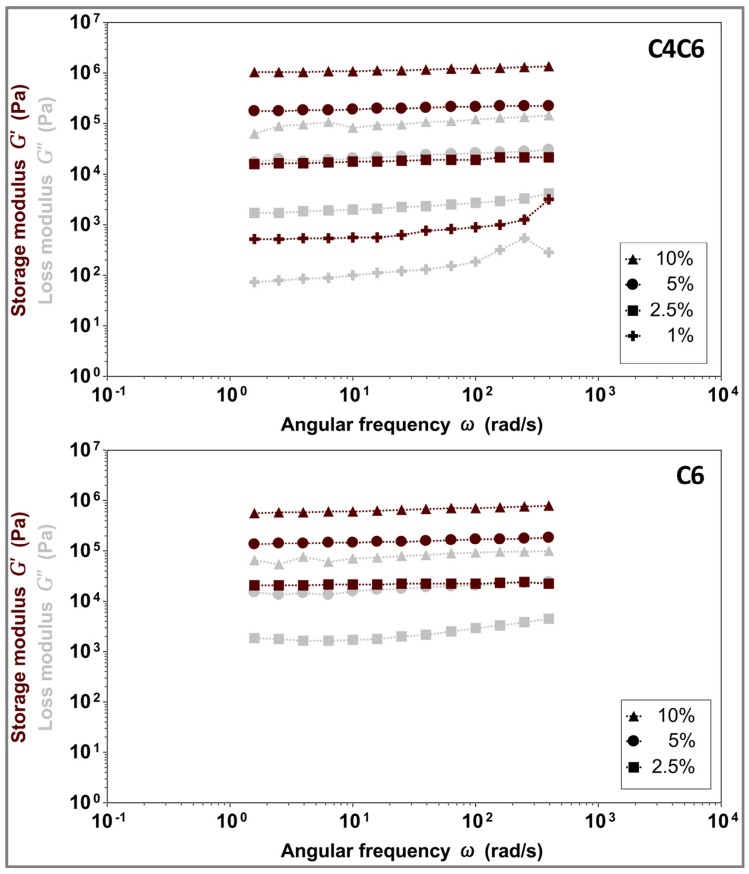
Concentration dependent viscoelastic properties of C4C6 (**top**) and C6 (**bottom**) hydrogels, indicating a gel-like behavior for all tested samples (G’ > G”). Maroon symbols indicate G’ (storage modulus), grey symbols indicate G” (loss modulus).

The stability of the hydrogels and their viscoelastic properties (storage modulus (G’) and loss modulus (G”)) were determined via oscillatory frequency sweeps (Figure 5). Both C4C6 and C6 hydrogels elicit gel-like behavior (G’ > G”) in the tested frequency range (1–400 rad/s, represented on the graph on a logarithmic scale). The G’ values are concentration dependent, and in the range of 27.6–492.5 kPa for C4C6 and 0.7–1117.6 kPa for C6, respectively. The C6 10% hydrogels were excluded from further testing as the polymer appeared to be incompletely solubilized and the materials appeared visually heterogeneous.

**Figure 6:**
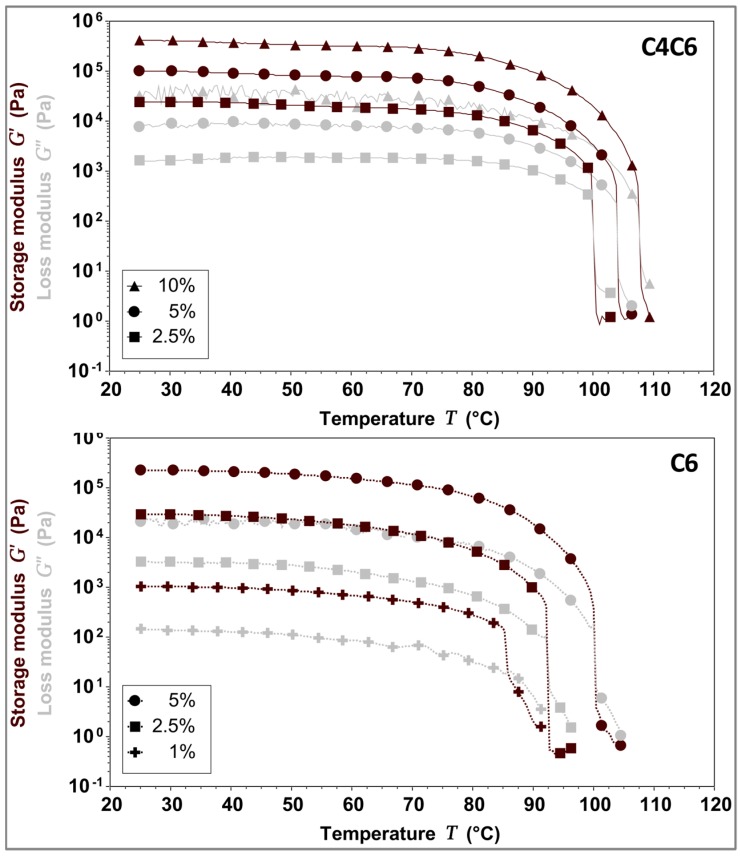
Gel-to-liquid transition temperature (*T*_G**→**__L_) determination for 5% *w/w* C4C6 (**top**) and 5% *w/w* C6 (**bottom**) hydrogels. C4C6 samples transitioned from a gel to a liquid state between 100–107 °C, while C6 sample transitioned from a gel to a liquid state between 85–101 °C, indicating that the nature of the diamine in the poly(glucaramide)s influences the structural stability of the hydrogels. No solvent loss occurred during the testing process. The experimental temperature range was 25–110 °C. Maroon symbols indicate G’ (storage modulus), grey symbols indicate G” (loss modulus).

Given that the polymers are solubilized at elevated temperatures and gel upon cooling, the temperature dependent behavior of C4C6 and C6 hydrogels was investigated. The data indicates that the gel-to-liquid transition temperature (*T*_G→L_) is concentration dependent. Furthermore, our results suggest that the doubling of the polymer concentration imparts a ~3 °C increase in the *T*_G→L_ for the C4C6 hydrogels, and an ~8 °C increase in the *T*_G→L_ for C6, respectively (Figure 6 and Table 3). 

**Figure 7:**
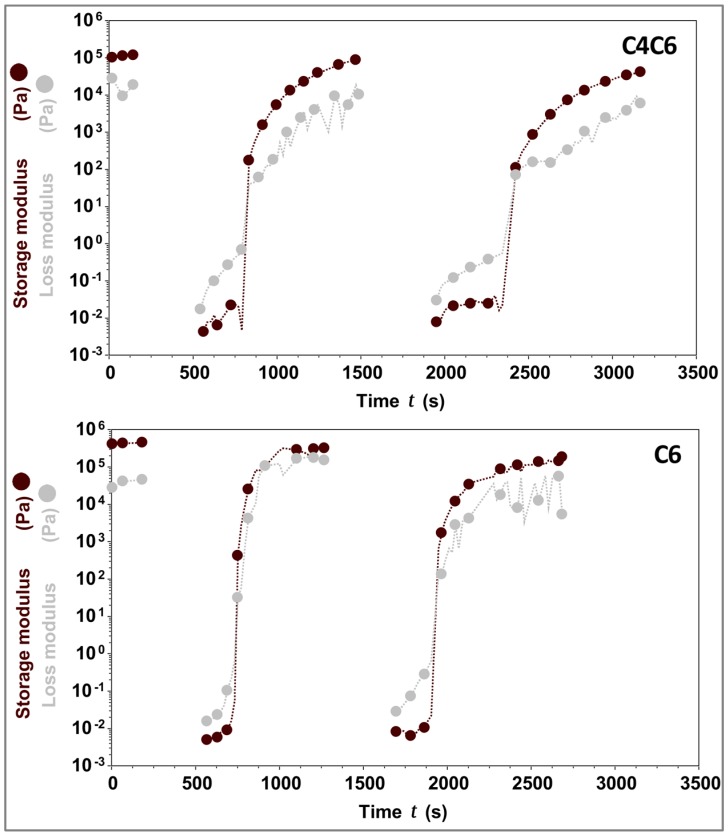
Thermoreversibility of C4C6 and C6 hydrogels. C4C6 (**top**) and C6 (**bottom**) were tested across three cycles of heating/cooling. The data indicates that upon cooling the solutions revert to gels with storage moduli similar to the initial values. The experimental temperature range was 25–110 °C. Maroon symbols indicate G’ (storage modulus), grey symbols indicate G” (loss modulus). The concentration of the hydrogels tested was 5% *w/w*.

Considering the effect of the temperature on the gelation process of these hydrogels, the potential thermoreversibility of these materials was next interrogated. For this, the gels (5% *w/w* C4C6 and 5% *w/w* C6) were liquefied at *T*_G**→**L_ + 3 °C, then rapidly cooled to 25 °C and analyzed rheologically. The data shows that both C4C6 and C6 hydrogels are capable of consistently reverting to a gel state with a G’ value identical or similar to the initial value (Figure 7).

Overall, the rheological data indicates that the poly(glucaramide)s form hydrogels with concentration dependent G’, *T*_G**→**L_, and that these hydrogels are capable of undergoing several heating/cooling cycles without significant changes in their mechanical responses. 

### 2.4. Controlled Release of STPP

Previously published data indicates that poly(glucaramide) breakdown products constitute a source of nitrogen that can be utilized by crops [25]. In this context, and in view of the micro- and macroscopic properties of the poly(glucaramide)-based hydrogels, we explored their performance as controlled release systems for fertilizer components, represented in our experiments by STPP. For these tests we only considered the aqueous based hydrogels (C4C6), as a more compatible system for plant nutrient targeted applications. The C6 hydrogel was therefore excluded, as it requires acetic acid for solubilization of the polymer. 

**Figure 8:**
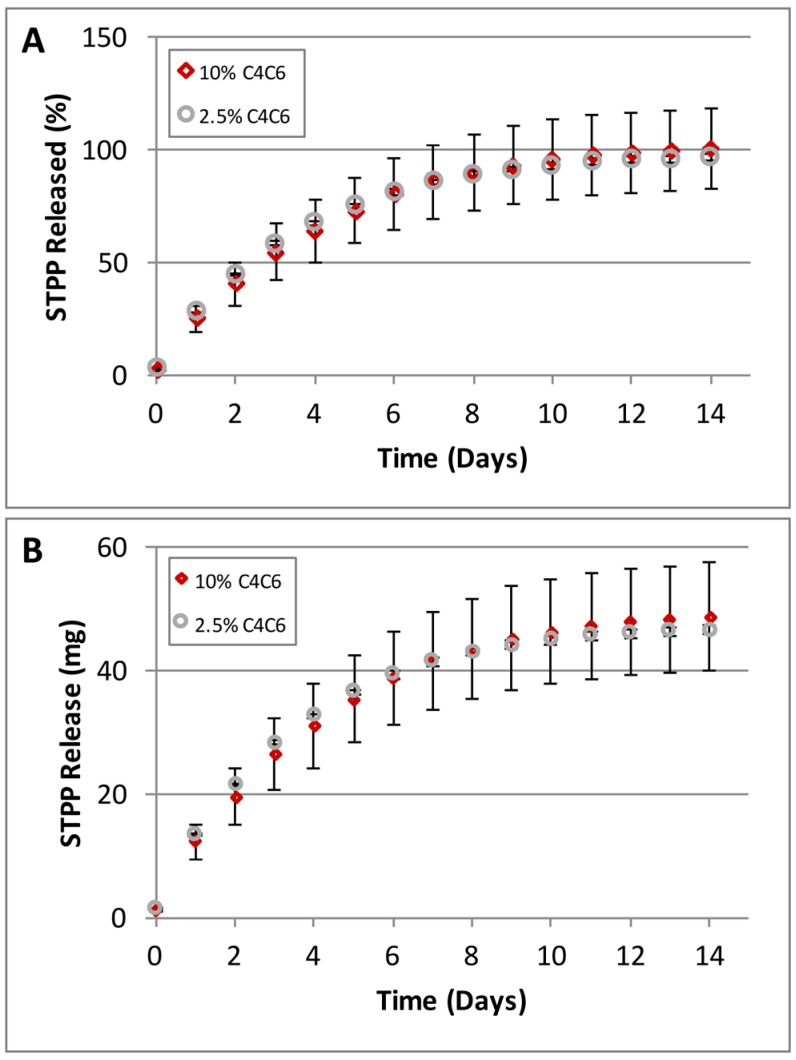
Slow release of Sodium Tripolyphosphate (STPP) from C4C6 hydrogels (the high error bars for the 10% hydrogel are due to the rapid gelation of the samples during casting, creating some inconsistencies). (**A**) STPP release rates represented as percent (%) released/day; (**B**) STPP released, represented as amount (mg) released/day. The data indicates that the C4C6 systems are capable of controlled release of small molecules.

C4C6 hydrogels obtained from 2.5% and 10% polymer solutions were loaded with 30 mg STPP/g gel and monitored for release for 14 days (Figure 8). The results show that the entrapped STPP is slow-released over a period of 14 days, with a more rapid rate in the first 5 days and diminishing after day 10 (Figure 8A). No differences were observed between the STPP amounts released from the 2.5% versus the 10% hydrogels (Figure 8B).

**Figure 9:**
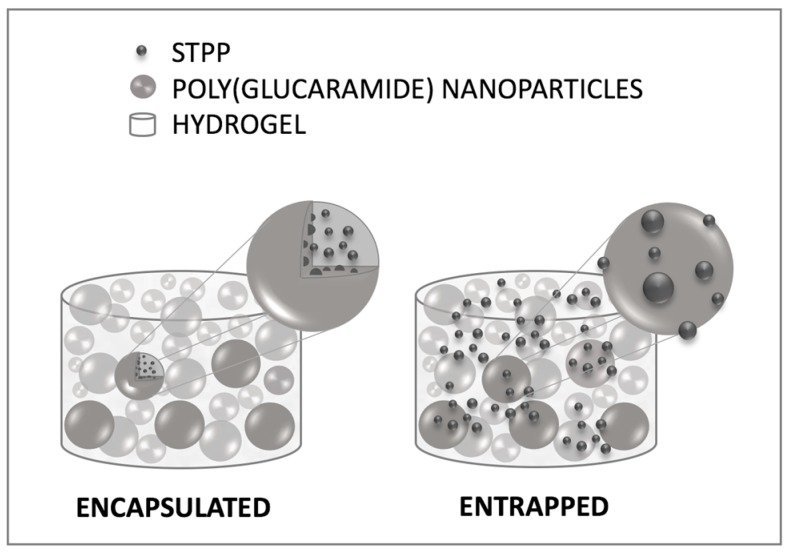
Illustration of potential STPP loading mechanisms in the poly(glucaramide) hydrogels with the small molecules either encapsulated in the nanoparticles (**right**) or entrapped in the hydrogel network (**left**).

Mechanistically, two different scenarios could be envisioned for the STPP loading: 1—the phosphate is encapsulated inside the nanoparticles; 2—the phosphate is outside the nanoparticles, entrapped at the surface or between the nanoparticles (Figure 9). To investigate the loading mechanism of STPP, hydrogels (2.5%) were loaded with different concentrations of phosphate (30–120 mg STPP/g gel), in an attempt to saturate the nanoparticles (Figure 9). The highest concentration of STPP used (120 mg/g gel) was limited by the molecule’s solubility. Our hypothesis was that, in the case of the first loading scenario—encapsulation—the addition of STPP concentrations exceeding the encapsulation capacity of the nanoparticles would result in a burst-type release of the phosphate. 

**Figure 10:**
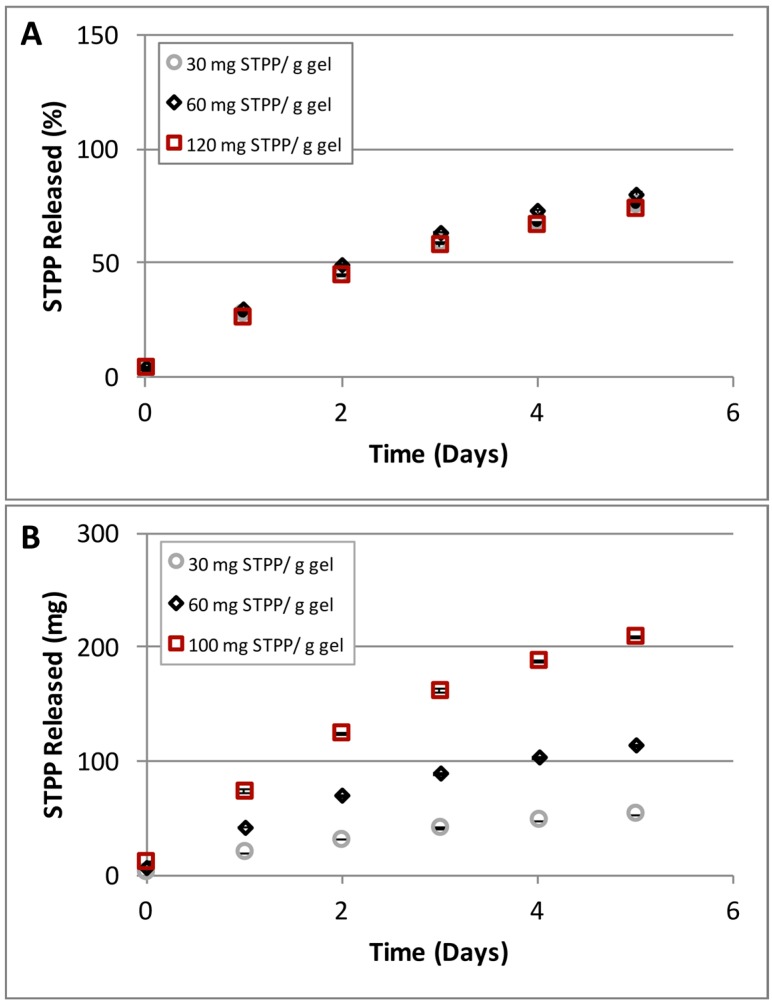
STPP concentration dependent release from C4C6 hydrogels. (**A**) hydrogels (2.5% *w/w*) loaded with different amounts of STPP (30, 40 and 120 mg STPP/g gel) showed identical release rates; (**B**) hydrogels (2.5% *w/w*) loaded with different amounts of STPP (30, 40 and 120 mg STPP/g gel) released the small molecule in amounts proportional to the loading concentration (the lowest amounts were detected for the 30 mg STPP/g gel while the highest released amounts were observed for the 120 mg STPP/g gel).

Our data shows no difference in the release rates for the STPP concentrations tested (Figure 10A). However the amounts of phosphate released correlated with the initial concentrations of STPP loaded (Figure 10B). Although there is the possibility that the STPP concentrations selected were not sufficiently high to exceed the encapsulation capacity of the gel constituent nanoparticles, taken together our data appear to indicate that STPP is encapsulated in the nanoparticles and released at a rate independent of the concentration of the initial polymer solution. While the STPP loading mechanism cannot be conclusively determined based on the above, our results clearly show that small molecules, as represented here by STPP, can be loaded and released in a controlled manner from the C4C6 poly(glucaramide)s hydrogels.

## 3. Conclusions

The aforementioned results describe the synthesis and characterization of GA-based poly(glucaramide)s, and their potential applicability as controlled release systems. We replicated a patented synthetic method to obtain poly(glucaramide)s via the condensation of GA with aliphatic diamines, formed poly(glucaramide)-based hydrogels, showed the hierarchical assembly of the hydrogel networks via the aggregation of nanoparticles, and the performance of these hydrogels as controlled release systems for small molecules such as STPP. Overall, this study further highlights the versatility of GA as a building block for the synthesis of sustainable materials, with potential for a wide array of applications. 

## 4. Materials and Methods

### 4.1. Reagents and Materials

Monopotassium d-glucarate was purchased from Applied Foods Sciences, LLC, (Austin, TX, USA). Sodium methoxide was purchased as a 0.5 M solution in methanol from Sigma-Aldrich (St. Louis, MO, USA). All other chemicals and solvents were purchased from Sigma-Aldrich (St. Louis, MO, USA) or J.T. Baker (Philipsburg, NJ, USA) and used without further purification. DOWEX 50WX8 ion exchange resin was obtained from Sigma Aldrich (St. Louis, MO, USA). 

### 4.2. Synthesis of Poly(glucaramide)s

Poly(glucaramide) hydrogels were prepared using a previously disclosed procedure [20]. Briefly, monopotassium d-glucarate (20.00 g, 80.57 mmol) was converted to its acid form using DOWEX 50WX8 ion exchange resin (70 mL, 147 mmol, Dowex 50WX8-100, 2.1 meq/mL). The selected diamine (84.1 mmol) was then added to the glucaric acid solution to form a stoichiometric diammonium d-glucarate salt, which was collected via precipitation in methanol (200 mL). Hexamethylene diammonium d-glucarate was isolated as a white powder (24.02 g, 73.60 mmol, 91.3%), and tetramethylene diammonium d-glucarate also yielded a white powder (21.45 g, 71.91 mmol, 88.9%). The diammonium d-glucarate salt or a combination of salts (7.02 mmol) was then reacted in methanolic HCl (prepared by the reaction of acetyl chloride (2.0 mL, 28 mmol) and methanol (45 mL)) to produce a mixture of methyl d-glucarate esters and glucaro-lactones. The resulting mixture was evaporated to dryness then redissolved in fresh methanol (20 mL) and neutralized with sodium methoxide solution to liberate the free amine and promote polycondensation. The polymerization reaction was carried out under ambient temperature and pressure, and the resulting polymeric precipitate was collected by vacuum filtration and dried. The synthesized polymers were designated according to the diamines used. Poly(hexamethylene d-glucaramide) designated C6, was isolated as a white powder (1.52 g, 5.24 mmol, 74.6%). Poly(tetramethylene d-glucaramide) designated C4, was isolated as a white powder (1.38 g, 5.26 mmol, 74.9%). Poly(tetramethylene/hexamethylene d-glucaramide) designated C4C6, was isolated as a white powder (1.43 g, 5.19 mmol, 74.0%). The degree of polymerization (DP) of the poly(glucaramide)s was determined by ^1^H-NMR. Specifically, the DP is estimated by comparing the distinct methylene signal adjacent to the terminal amine to the internal methylene signals adjacent to the amide groups. The ratio of the terminal amine methylene protons to half the amide methylene protons to gives the average number of repeating units for the polymer sample. The following DPs were determined: C4 5.9 (MW ~ 1550 g/mol), C4C6 5.6 (MW ~ 1550 g/mol) and C6 6.3 (MW ~ 1800 g/mol). 

### 4.3. Preparation of Poly(glucaramide) Hydrogels

Hydrogels were prepared by dissolving different amounts of the poly(glucaramide)s in the appropriate solvent (water for C4C6 and 20% acetic acid in water for C6), heating them to boiling, then allowing the solution to cool to promote gel formation. The water soluble C4 polymers do not form hydrogels. The C4C6 polymers are partially water soluble, and for certain concentrations (up to 10% *w/w*) total water solubility could only be achieved at elevated temperatures (boiling). Acetic acid was used as co-solvent to promote the solubilization of the water insoluble C6 sample. Specifically, for hydrogel formation, C6 polymers were dissolved in a 20% *v*/*v* acetic acid solution at the desired polymer concentration. This allowed for the C6 polymer concentration to be comparable to the water soluble (C4 and C4C6) samples.

### 4.4. Hydrogel Characterization

#### 4.4.1. Scanning Electron Microscopy (SEM)

The internal structures of the hydrogels were investigated with a Hitachi S-4700 Field Emission scanning electron microscope (Hitachi Instruments Inc., Pleasanton, CA, USA). The hydrogel samples were prepared by allowing 250 μL of polymer solution to gel in a 24 well plate. These gels were washed 3 times with 0.5 mL of deionized water for 10 min each. The samples were placed in a freezer (−20 °C) overnight and then lyophilized. The dried hydrogel samples were placed on a conductive carbon tab on an aluminum SEM stub. Samples were sputter coated with gold in a Denton Desk V sputter coater (Denton Vacuum LLC, Moorestown, NJ, USA) and then imaged.

#### 4.4.2. Dynamic Light Scattering (DLS)

To confirm the particulate nature of the polymers in a hydrated state poly(glucaramide) solutions were investigated at 25 °C using a Zetasizer nano ZS DLS spectrometer (Malvern Instruments Ltd., Malvern, UK). The DLS experiments were performed on poly(glucaramide) solutions in deionized water passed through a 0.22 µm filter (Merck Millipore Ltd., Tullagreen, Carrigtwohill, Co. Cork, Ireland). Particle size distributions were determined at time 0, 1 and 3 h.

#### 4.4.3. Rheological Characterization

Rheological measurements were obtained with a Discovery HR2 hybrid rheometer (TA Instruments, New Castle, DE, USA) and a 20 mm diameter parallel plate fixture. All hydrogels were characterized with a 1.00 mm gap and within the materials determined pseudo-linear viscoelastic range. All rheological characterizations were completed at 25 °C unless otherwise specified. Oscillatory stress sweeps were conducted on the hydrogels to determine the pseudo-linear viscoelastic region. These measurements were completed with a stress range of 0.0064–640 Pa with a frequency of 1 Hz. Oscillatory frequency sweeps were conducted to determine the hydrogel’s response to a range of frequencies. These measurements were completed with an angular frequency range of 1 to 400 rad/s with constant stress. An oscillatory temperature ramp was employed to investigate the hydrogels gel-to-liquid transition properties. The temperature was controlled through a Peltier plate and the instrument was calibrated to compensate for expansion during the heating process to maintain the specified gap. Temperature sweeps were conducted between 25–110 °C with a ramp rate of 5 °C/min and with constant stress and a frequency of 1 Hz. To prevent the loss of solvent at increased temperatures a low viscosity oil was applied to the outer surface of the hydrogels samples once the geometry gap was achieved. This allowed for the accurate rheological characterization of the poly(glucaramide) hydrogels at elevated temperatures without concerns of solvent loss. The gel-to-liquid transition temperatures were determined by calculating the intersection of the storage and loss modulus (G’ and G”, respectively). The thermoreversibility points of the hydrogel samples were examined by loading a hydrogel sample and first determining its dynamic shear modulus at 25 °C. The temperature of the system was then raised to 5 °C above the gel-to-liquid transition temperature of the hydrogel. Samples were allowed to equilibrate for 3 mins after which the system was returned to 25 °C and the dynamic shear moduli were monitored as a function of time. Gelation was considered to have occurred when G’ > G”. Hydrogels were allowed to set until similar dynamic shear moduli to the initial sample were seen, and then the analysis was repeated. The thermoreversibility characterization was carried out at a lower constant stress and frequency to allow for the accurate measurement of the dynamic shear moduli without over disturbance of the liquid/hydrogel samples, which could inhibit gel formation.

### 4.5. Sodium Tripolyphosphate (STPP) Release Studies

The C4C6 polymer was dissolved in a solution of STPP solution to achieve the desired STPP and polymer concentrations. The polymer and STPP solution was separated into three individual 2.5 g samples and allowed to gel overnight. The gelled samples were then washed twice with 2.0 mL of deionized water and the wash solution was collected and analyzed by IC-MS to determine the STPP concentration. The subsequent release of STPP was monitored by the addition of deionized water (2.0 mL) to the top of the gel mixture and collection of the liquid phase after 24 h of exposure to the gel surface. The addition and collection was repeated daily for two weeks, and STPP release was quantified using IC-MS. Total STPP released was calculated by adding each daily release to the previous total released and percent released was calculated by dividing the total STPP measured by the theoretical STPP added to the samples. Error bars and averages were calculated with an *n* = 3.

## Figures and Tables

**Table 1:** Compilation of the water solubility and gel forming properties of selected poly(glucaramide)s.

Poly(glucaramide)	Water Solubility	Gel Formation	Minimum Gelling Concentration (MGC, % *w*/*w*)	Solvent
Poly(tetramethylene d-glucaramide) (**C4**)	Yes	No	N/A	N/A
Poly(tetramethylene/hexamethylene d-glucaramide) (**C4C6**)	Partial	Yes	2.5%	Water
Poly(hexamethylene d-glucaramide) (**C6**)	No	Yes	1%	Acetic acid (20% *v*/*v*)

**Table 2:** Time-dependent particle size distributions of poly(glucaramide) solutions.

Time (h)	C4		C4C6		C6	
	Particle Size (Diameter, nm)	Intensity * (%)	Particle Size (Diameter, nm)	Intensity * (%)	Particle Size (Diameter, nm)	Intensity * (%)
0	4.3 ± 1.6 148.4 ± 83.8 4065 ± 1060	76.6 22.3 1.0	4.5 ± 1.4 218.1 ± 118.1 4468 ± 897.9	65.8 33.2 1.0	3.9 ± 1.6 4429 ± 921.3	94.8 5.2
1	4.3 ± 1.8 124.8 ± 55.6 5032 ± 593.8	75.2 21.8 3.0	4.1 ± 1.1 387.2 ± 361.2 3709 ± 1061	60.4 37.6 2.0	3.8 ± 0.8 2304 ± 382.2	18.4 81.6
3	4.3 ± 1.6 175.4 ± 97.4	78.8 21.2	4.1 ± 1.3 68.2 ± 28.5 700.2 ± 412.4	40.7 4.9 54.4	ND	ND

* The intensity output correlates with the population distribution of the particles detected. ND = Not detected in the analyzed range.

**Table 3:** Gel-to-liquid transition temperature (*T*_G**→**L_) for the C4C6 and C6 hydrogels. Samples excluded from testing were C4C6 1% *w/w* (concentration is below the minimum gelling concentration) and C6 10% *w/w* (starting solution appeared to contain undissolved polymer).

Polymer Concentration (% *w/w*)	T_G__→L_ (°C)	
	C4C6	C6
1	Not tested	85.5 ± 0.5
2.5	100.4 ± 0.7	93.0 ± 0.7
5	103.7 ± 0.3	101.0 ± 1.4
10	107.3 ± 0.5	Not tested

## References

[1] Ahmed E.M. (2015). Hydrogel: Preparation, characterization, and applications: A review. J. Adv. Res..

[2] Li J., Mooney D.J. (2016). Designing hydrogels for controlled drug delivery. Nat. Rev. Mater..

[3] Ullah F., Othman M.B., Javed F., Ahmad Z., Akil H. (2015). Classification, processing and application of hydrogels: A review. Mater. Sci. Eng. C Mater. Biol. Appl..

[4] Vashist A., Ahmad S. (2015). Hydrogels in tissue engineering: Scope and applications. Curr. Pharm. Biotechnol..

[5] Du X., Zhou J., Shi J., Xu B. (2015). Supramolecular hydrogelators and hydrogels: From soft matter to molecular biomaterials. Chem. Rev..

[6] Highley C.B., Prestwich G.D., Burdick J.A. (2016). Recent advances in hyaluronic acid hydrogels for biomedical applications. Curr. Opin. Biotechnol..

[7] Kyburz K.A., Anseth K.S. (2015). Synthetic mimics of the extracellular matrix: How simple is complex enough?. Ann. Biomed. Eng..

[8] Seliktar D. (2012). Designing cell-compatible hydrogels for biomedical applications. Science.

[9] Huang W., Rollett A., Kaplan D.L. (2015). Silk-elastin-like protein biomaterials for the controlled delivery of therapeutics. Expert Opin. Drug Deliv..

[10] Sood N., Bhardwaj A., Mehta S., Mehta A. (2016). Stimuli-responsive hydrogels in drug delivery and tissue engineering. Drug Deliv..

[11] Alge D.L., Azagarsamy M.A., Donohue D.F., Anseth K.S. (2013). Synthetically tractable click hydrogels for three-dimensional cell culture formed using tetrazine-norbornene chemistry. Biomacromolecules.

[12] Mironi-Harpaz I., Wang D.Y., Venkatraman S., Seliktar D. (2012). Photopolymerization of cell-encapsulating hydrogels: Crosslinking efficiency versus cytotoxicity. Acta Biomater..

[13] Kim M.H., Park W.H. (2016). Chemically cross-linked silk fibroin hydrogel with enhanced elastic properties, biodegradability, and biocompatibility. Int. J. Nanomed..

[14] Moon T.S., Dueber J.E., Shiue E., Prather K.L.J. (2010). Use of modular, synthetic scaffolds for improved production of glucaric acid in engineered *E. coli*. Metab. Eng..

[15] Smith T.N., Hash K., Davey C.L., Mills H., Williams H., Kiely D.E. (2012). Modifications in the nitric acid oxidation of d-glucose. Carbohydr. Res..

[16] Thaburet J.F., Merbouh N., Ibert M., Marsais F., Queguiner G. (2001). Tempo-mediated oxidation of maltodextrins and d-glucose: Effect of ph on the selectivity and sequestering ability of the resulting polycarboxylates. Carbohydr. Res..

[17] Kiely D.E., Hash K.R. (2010). Method of oxidation using nitric acid. U.S. Patent.

[18] Werpy T., Petersen G. (2004). Top Value Added Chemicals from Biomass: Volume I—Results of Screening for Potential Candidates from Sugars and Synthesis Gas.

[19] Kiely D.E., Chen L., Lin T.H. (1994). Hydroxylated nylons based on unprotected esterified d-glucaric acid by simple condensation-reactions. J. Am. Chem. Soc..

[20] Kiely D.E., Smith T.N. (2014). Hydroxypolyamide Gel Forming Agents. U.S. Patent.

[21] McBeath T.M., Lombi E., McLaughlin M.J., Bunemann E.K. (2007). Polyphosphate-fertilizer solution stability with time, temperature, and pH. J. Plant Nutr. Soil Sci..

[22] Perez J.J., Francois N.J. (2016). Chitosan-starch beads prepared by ionotropic gelation as potential matrices for controlled release of fertilizers. Carbohydr. Polym..

[23] Tyliszczak B., Polaczek J., Pielichowski J., Pielichowski K. (2009). Preparation and Properties of Biodegradable Slow-Release PAA Superabsorbent Matrixes for Phosphorus Fertilizers. Macromol. Symp..

[24] Villalba G., Liu Y., Schroder H., Ayres R.U. (2008). Global phosphorus flows in the industrial economy from a production perspective. J. Ind. Ecol..

[25] Lampila L.E. (1993). Functions and uses of phosphates in the seafood industry. J. Aquat. Food Prod. Technol..

[26] Dodds W.K., Bouska W.W., Eitzmann J.L., Pilger T.J., Pitts K.L., Riley A.J., Schloesser J.T., Thornbrugh D.J. (2009). Eutrophication of us freshwaters: Analysis of potential economic damages. Environ. Sci. Technol..

[27] Jahns T., Kiely D.E. (2006). Abiotic hydrolysis of some poly-d-glucaramides and subsequent microbial utilization/degradation. J. Polym. Environ..

